# The association between adolescents’ self-esteem and perceived mental well-being in Sweden in four years of follow-up

**DOI:** 10.1186/s40359-023-01450-6

**Published:** 2023-11-25

**Authors:** Kristina Carlén, Sakari Suominen, Lilly Augustine

**Affiliations:** 1 School of Health Sciences, University of Skövde, Box 408, Skövde, 54128 Sweden; 2 Department of Public Health, University of Turku, Turku, Finland; 3 School of Learning and Communication, Jönköping University, Jönköping, Sweden

**Keywords:** Adolescence, Mental well-being, Promotion, Self-esteem, Self-concept

## Abstract

**Background:**

The situation concerning adolescent mental health is a global public health concern, and the concept includes the ability to cope with problems of everyday life. A person’s approach and attitude towards themselves, i.e., their self-esteem, affects mental health. The study aimed to appraise and deepen the scientific understanding of adolescents’ self-reported self-esteem at age 12−13 from a resource perspective and test its ability to predict subsequent perceived mental well-being at age 17.

**Methods:**

Data from the Longitudinal Research on Development in Adolescence (LoRDIA) prospective follow-up study of adolescents aged 12−13, and 17 (*n* = 654) were analysed using ANCOVA. The outcome variable, perceived mental well-being (MWB), covers the aspects of mental well-being inspired by the “Mental Health Continuum,” representing positive mental health. Covariates were self-esteem (SE) and reported initially perceived MWB at age 12−13. Other independent explanatory variables were gender, the family’s economy, and the mother’s educational level.

**Results:**

Self-esteem appeared relatively stable from 12−13 to 17 years (M = 20.7 SD = 5.8 vs. M = 20.5 SD = 1.7). There was a significant but inverted U – shaped association between SE at age 12–13 and perceived MWB at age 17 [F (1, 646) = 19.02, β-0.057; CI -0.08−-0.03, Eta = 0.03, *p* = .000]. Intermediate but not strong SE predicted significantly good MWB. When conducting the ANCOVA for boys and girls separately, only the mother’s educational level was significantly positively associated with perceived MWB of girls.

**Conclusions:**

Good self-esteem in early adolescence increases the likelihood of an unchanged favourable development of self-esteem and the probability of good perceived mental well-being. SE explained 18 per cent of the variation of MWB, and even more among girls. However, normal SE rather than high SE at 12 and 13 years is predictive of later mental well-being. Girls reported low self-esteem more often. Therefore, supporting self-esteem early in life can promote mental well-being in adolescence.

**Supplementary Information:**

The online version contains supplementary material available at 10.1186/s40359-023-01450-6.

## Background

Adolescent mental health is a global public health concern and needs prioritisation [1]. Besides having direct effects, mental health also has indirect effects that play a major role, as concurrent mental health problems also affect the next generation [2]. Good mental health is a personal asset that includes the ability to cope with everyday problems. According to the theory of salutogenesis [3], successful coping implies perceiving stressors as comprehensible, manageable, and meaningful. Good mental health enables individuals to become aware of their abilities and contribute to the community through productive and fruitful work and functioning [4]. However, mental health is a broad concept consisting of both mental well-being and mental ill health, including mental disorders [5]. As the WHO [1] has stated, over half of all cases of mental disorders debut by age 14, and most cases remain untreated well into adulthood. Meanwhile, the global prevalence of mental disorders among children and adolescents was 13.4 per cent (CI 11.3–15.9) [6], which underlies the importance of a promotive perspective.

Self-esteem involves an approach and attitude towards oneself that is either global or more specific [7]. Global self-esteem, in turn, has two dimensions: self-competence, indicating the evaluative experience of oneself as a causal agent, and further self-liking, the experience of oneself as a valuable social object, a good or bad person who, in the former case, can contribute to group harmony [8]. Global self-esteem is more closely connected to psychological well-being than emotional well-being. In contrast, specific self-esteem (such as academic self-esteem, which deals with the perception of learning opportunities) connects to behaviour and school performance [7]. Self-esteem consists of perceptions of what one thinks of oneself and individuals’ negative or positive attitudes towards themselves [9]. Later research [10] has shown that self-esteem could also be seen in terms of self-worth, suggesting three dimensions of esteem: worth-based, efficacy-based, and authenticity-based. These dimensions primarily emerge through verification of the social environment, from the group about the role, and from personal identities. Hence, self-esteem naturally affects mental health [11] and arguably should relate to later mental well-being, and therefore needs to be tested.

The theoretical base for the variable of mental well-being is the mental health continuum [12], consisting of emotional, social, and psychological well-being [13]. Psychological well-being includes an experience of purpose and meaning in life [13]. Self-esteem and its relation to mental health according to Keyes [12] has not been investigated before. While Keyes’ concept measures three dimensions, our study will focus on mental well-being, i.e. psychological and emotional well-being but without inclusion of its social sub-component.

Adolescence is the period in life when most developmental changes take place within a relatively short period. During this period, self-esteem generally fluctuates to a low level in childhood but increases throughout adolescence and young adulthood [14]. Therefore, self-esteem also represents an essential psychological asset that can be scaffolded to facilitate mental health over a lifespan. High self-esteem is associated with subjective well-being [15], a measure of subjective quality of life [16], which does not coincide with well-being [15] or mental well-being per se without including quality of life. The relationship between self-esteem and well-being is not always straightforward; high self-esteem can also be associated with narcissism or aggressive behaviour, especially in boys [11]; at the same time as low self-esteem can be related to reactive aggression [17]. People with a broad range of mental disorders, such as anxiety, depression, and suicidal ideation, also report low self-esteem [18]. In addition, people with externalising problems report low self-esteem (e.g. violence, substance use, and risk behaviour), social problems like poor social functioning, and show school dropout [11]. Therefore, studies looking at the relationship between self-esteem and later mental well-being in terms of perception of health and purpose are necessary, as self-esteem can be supported by external interventions.

Well-being as a concept relates to mental health. Mental health as well as self-esteem shows a gender bias: girls tend to report more emotional problems [19] and lower self-esteem [20] than boys. Therefore, gender should affect the association between self-esteem and mental health. While positive attitudes towards oneself support mental health [21], attitudes are related to social roles and expectations. In addition to gender, other factors exerting their influence mainly via societal mechanisms—such as shaping experiences, attitudes, and expectations—affect mental health [22]. Such factors are represented, for instance, by the economic and educational situation of the family, especially the mother’s educational level. Most previous research has focused on mental health risk factors with different mental disorders as the outcome [18]. In contrast, the present study aims to apply a resource perspective in line with the practical and clinical need to focus on well-being in adolescence instead of seeking methods to reduce mental ill health symptoms in adolescence [23]. Furthermore, this aligns with the need for follow-up mental and health studies [24]. Based on this, health promotion, enabling people to increase control over and improve their health [25], could then support adolescents’ mental health [26]. Specifically, the study aimed to appraise and deepen the scientific understanding of adolescents’ self-reported self-esteem at age 12−13 from a resource perspective and test its ability to predict subsequent perceived mental well-being at age 17.

## Methods

### Study design and setting

The Longitudinal Research in Development in Adolescence (LoRDIA) study started in 2013, following adolescents aged 12−13 to 17 in four municipalities in Southwestern Sweden and inviting the total population of sixth- and seventh-grade adolescents to participate. The participants (*n* = 1884) consisted of almost equal shares of boys (50.7%; *n* = 956) and girls (49.3%; *n* = 928). The research team visited the four municipalities to distribute and collect the survey responses in class.

### Participants

Data collection started in 2013 (Wave 1 = W1); adolescents aged 12 and 13 agreed to participate, a total of 1515 children. At 17 years of age, the fifth wave of LoRDIA was collected, and 949 children participated. A total of 654 individuals responded to both waves for this particular study’s relevant waves. A published report describes the LoRDIA planning and participants on p. 44–45 [27].

### Instruments

#### Rosenberg’s Self-Esteem (SE) scale

A scientifically well-known and frequently used instrument [28] validated in Swedish [29]. The scale consists of ten items—such as “I am pleased with myself”, “Sometimes I think that I’m not capable of anything” or “I often feel like a failure”—with a four-grade Likert scale ranging from “No, I don’t agree at all” to “Yes, I agree” (0–3 points). The scale ranges from 0 to 30, and a score of 25 or higher suggests high self-esteem; scores between 15 and 25 are considered to be within the normal range, and scores less than 15 indicate low self-esteem [28]. Two different variables were created based on the SE scale. Self-esteem was categorized in three steps, low, normal, and high; this categorization was used for descriptive statistics. A sum variable based on the total sum (range 3–30) is used when a continuous SE variable is needed, e.g. in the ANCOVA model.

#### Perceived Mental well-being (MWB)

A variable assessing perceived mental well-being was created to test the relationship between positive mental health and self-esteem. The scale for perceived mental well-being (MWB) comprises a sum score from three questions: “How healthy do you think you are?”, “How do you feel about life right now?”, and “I think my life has purpose and meaning.” In W1, the response scale was a 3-grade rating scale from “very healthy to not healthy at all”, but in W5, a 4-grade rating scale ranging from “totally healthy, quite healthy, not particularly healthy, and not healthy at all” was used. For the second question: “how do you feel about life right now?“ in W1, the response scale started from “very good and ranged to not particularly good”. In W5, the response scale varied from “very good, pretty good, not particularly good, and not good at all”. For the third question “I think my life has purpose and meaning”, the response scale in W1 ranged from “yes to no” and finally in W5 as follows: “totally agree, partially agree, takes a partial distance, and takes distance completely”. When adding the three questions as a sum score, Cronbach’s α was .715 in W1 and .719 in W5, indicating high internal consistency in measuring positive mental health. The variables were recoded into high and low MWB in W1 as follows: 1 = very healthy, 0 = quite healthy, and not healthy. Corresponding for W5 was: 1 = very healthy, and 0 = all other alternatives. By handling each question this way, the sum score for MWB using the three questions varied from 0 to 3, indicating low, normal, or high positive mental health. Higher scores stand for better mental health. Categories described the groups, but the total sum was used in the forthcoming ANCOVA analyses as the outcome. In Wave 1, the range of the total sum was 0−6, and in Wave 5, 0−9.

#### Background variables

Gender, the mother and father’s educational levels (secondary vs. post-secondary education), and the family’s economy are relevant background variables. The variable of the family’s economy comprised three questions: “The family’s income in comparison with other families”, “The family’s income in comparison to other classmates”, and “I can afford things that are expensive to me.” To every question, three levels of answers were applicable: “worse”, “medium”, or “high” (0–2). The Cronbach’s α for the sum score of the family’s economy was .617, its range was 0–6, and it was divided into three categories: worse (0–2), medium (3–4), and high (5–6). All of these variables are fixed factors in the ANCOVA.

### Statistical methods

Self-rated SE and perceived MWB at 12–13 and 17 years of age were tested using an independent t-test between groups divided into gender and a paired sample t-test for the dependent association over time. Categorical descriptions were made using Chi-square and cross-tables, and gender differences in the MWB and SE categories were investigated.

A general linear analysis of Covariance (ANCOVA) was conducted using the continuous variable self-esteem (SE) at age 17 as the outcome and concomitantly including the continuous sum scores SE and perceived MWB as covariates at age 12–13. The criteria for modelling normality and homogeneity via Levene’s test (*p* = .098) and using a random independent sample were fulfilled. The association was also checked for independent background variables, such as gender, the mother’s educational level, and the family’s economy.

To analyse the relation between SE and subsequent perceived MWB, a general linear model, ANCOVA was used in several steps. The dependent continuous variable perceived MWB at 17 years as an outcome. First (a), the association of initial perceived MWB at age 12−13 (W1) with the outcome of MWB four years later (W5) was calculated in a univariate model. In a second univariate model (b), initial perceived MWB was replaced with initial SE at age 12−13 (W1) as a continuous variable. In a third model (c), these two covariates (SE and perceived MWB at age 12−13) were used in a multivariable model. The criteria for conducting the variance models, ANCOVA, normality, homogeneity of variance via Levene’s test (*p* = .319), and using a random independent sample were fulfilled. Background variables (independent variables, handled as fixed factors) which were gender, mother’s educational level, and family´s economy, were used as categorical variables at age 12−13, chosen from previous research [19, 22]. The dataset was then split by gender to find associations between gender concerning SE and MWB. The significance level was set at *p* < .05.

SE in those participating only in Wave 1, *n* = 1472, vs. those participating in both waves, *n* = 936, and perceived MWB with the corresponding figures *n* = 1414 vs. *n* = 948 was compared. No significant difference was detected when comparing the mean values using an independent t-test for those participating in one versus two waves. The results are presented using descriptive statistics and variance models, including the β and F-values. SPSS version 27 was used for all statistical analyses.

## Results

While the initial sample had an even distribution of boys and girls, those participating in both waves included more girls than boys (*n* = 654, 57% girls), indicating significantly more attrition in boys. Both SE and perceived MWB were stable over time. However, there was a slight and significant decrease in SE (see Tables 1 and 2).

**Table 1:** Self-esteem (SE) and perceived mental well-being (MWB) between boys and girls at ages 12−13 and 17

	12-13 years	Meandiff	t-test between gender	17 years	Meandiff	t-test between gender
**Self-esteem**	Mean (SD) n			Mean (SD) n		
Total	20.7 (5.8) 1472	-2.168	-7.30**	20.5 (6.6) 936	-2.712	-6.41**
Girls	19.6 (6.2) 753			19.3 (6.4) 522		
Boys	21.8 (5.2) 719			22.0 (6.5) 414		
**Mental well-being**	Mean (SD) n			Mean (SD) n		
Total	4.4 (1.5) 1414		6.0**	4.9 (1.7) 948		2.9*
Girls	4.6 (1.5) 717		.457	5.1 (1.6) 528	.316	
Boys	4.1 (1.3) 697			4.7 (1.7) 420		

*p*<.005 * *p*<.001**

**Table 2:** Self-esteem (SE) and perceived mental well-being (MWB) according to participation in different waves

Paired t-test		Mean (SD)	Correlation	Mean difference (SD)	t
Pair 1	SE W1	20.82 (5.6)	.457**	.273 (6.4)	1.194
*n*=779	SE W5	20.55 (6.5)			
Pair 2	MWB W1	4.32 (1.4)	.380**	-.594 (1.7)	-9.417
*n*=746	MWB W5	4.91 (1.6)			

Self-esteem (SE) and perceived mental well-being (MWB), mean and mean differences and correlations at ages 12-13 and 17 for both girls and boys according to participation in different waves (W1, W5) of follow-up

*p*<.005 *, *p*<.001 **

The correlation between SE at 12−13 and 17 years) showed a stable trend during adolescence (r^2^ = .21).

It was more than twice as common for girls to have low SE than boys at both ages (see Table 1). Regarding SE, boys had about 1.5 times higher SE than girls, which is not as significant at age 17. It was almost twice as common for girls to have low MWB than boys; the percentage increased for boys and girls up to 17 years, but the difference was relatively almost as high (for more details, see Table 3).

**Table 3:** Gender differences and self-esteem (SE) perceived mental well-being (MWB) at ages 12−13 and 17

	SE at W1 (12-13 years) *n*=1472	SE at W5 (17 years) *n*=936	MWB at W1 (12-13 years) *n*=1414	MWB at W5 (17 years) *n*=948
	% (n)	% (n)	% (n)	% (n)
**Low**				
Total	13.3 (196)	19.7 (184)	11.4 (246)	27.6 (262)
Girls	9.3 (137)	13.1 (123)	11.2 (159)	17.3 (164)
Boys	4.0 (59)	6.5 (61)	6.2 (87)	10.3 (98)
**Normal/typical**				
Total	63.9 (941)	53.7 (503)	44.6 (631)	48.2 (457)
Girls	32.7 (482)	31.4 (294)	23.2 (328)	27.2 (258)
Boys	31.2 (459)	22.3 (209)	21.4 (303)	21.0 (199)
**High**				
Total	22.8 (335)	26.6 (249)	38.0 (537)	24.2 (229)
Girls	9.1 (134)	11.2 (105)	16.3 (230)	11.2 (106)
Boys	13.7 (201)	15.4 (144)	21.7 (307)	13.0 (123)
**Gender difference in all groups Chi** ^**2**^	44.24**	29.29**	33.91**	15.73**

Gender differences regarding low, typical, and high self-esteem (SE) perceived mental well-being (MWB) at ages 12-13 (W1) and 17 (W5) for girls and boys according to participation in different waves (W1, W5) of follow-up

*p*<.005 *, *p*<.001 **

Variables significantly associated with SE at age 17 were gender, initial family’s economy, SE, and perceived MWB at 12−13. Four years later, a lower family economy at W1 was associated with a higher SE (Table 4). Girls had significantly lower SE at age 17 than boys, even when including SE at age 12−13 as a covariate. Previous MWB also negatively impacted future SE; for every unit with higher SE, perceived MWB decreased by 0.54 units.

**Table 4:** Variables associated with self-esteem (SE) at 17 years

Variables associated with SE at 17 years of age ***n***=936						
**Independent variables**	**β**	**t**	**(df), F**	**CI**	**η** ^**2**^	**R** ^**2**^
Gender Girls	2.086**	-4.64	21.555	-2.97--1.20	**.032**	
Boys	**0**					
The Mother’s educational						
level Post-secondary	-0.989	-1.88	3.542	-2.02 – 0.04	.005	
Secondary	0					
The Father’s educational						
level Post-secondary	0.097	0.19	0.035	-0.92-1.11	.000	
Secondary						
The family’s economy						
worse	-1.665*	0.77	2.543	-3.18 – -0.15	**.007**	
medium	-0.879	-1.67		-1.91 – 0.15	.004	
high	0					
SE 12-13 years	0.400**	8.00	(1,644) 64.037	0.30 – 0.50	**.090**	
MWB 12-13 years	-0.538*	-2.70	(1,644) 7.269	-0.93 – -.15	**.011**	
						.27

Variables associated with self-esteem (SE) at 17 years of age in a multivariable ANCOVA model, i.e., gender, parental education, the family’s economy, perceived mental well-being (MWB), and initial self-esteem (SE) at age 12-13

*p*<.005*, *p*<.001**

Three ANCOVAs were conducted to determine the association with perceived MWB at 17 years: in Step a, using perceived MWB at age 12−13 as a covariate; in Step b, SE at age 12−13, but not MWB at age 12−13, were used; in Step c, both covariates were used (Table 5). In Model a, with initial perceived MWB as a covariate, the weak family´s economy positively predicts future MWB. In Model b, this relation remains despite using initial SE at age 12−13 as a covariate instead. In Model c, using both covariates, we see that these variables explain future perceived positive MWB, but the family´s economy no longer yields a significant association. Using both covariates explains more than using just one. The R^2^ increased to.18 from .15 in the model a and .14 in the model b. Eta^2^ for both covariates indicates medium to large effects on future perceived MWB.

**Table 5:** Summary of a hierarchical ANCOVA model for variables predicting perceived mental well-being (MWB) at 17 years

**a) MWB at 12–13 years of age as a covariate ***n*** = 661**						
	β	t	(df), F	Cl	η^2^	R^2^
Gender Girls Boys	0.085 0	0.717	0.514	-0.15-0.32	0.001	
Mother’s educational level Post-secondary Secondary	0.087 0	0.623	0.388	-0.19–0.36	0.001	
Father’s educational level Post-secondary Secondary	0.168 0	1.236	1.527	-0.10-0.44	0.002	
The family’s economy worse medium high	0.461* 0.126 0	2.276 0.904	2.642	0.06–0.86 − 0.15–0.40	**0.008** 0.001	
MWB at 12–13 years	0.399**	**9.362**	**(1,654)** **87.639**	**0.32–0.48**	**0.118**	
						.15
**b) Self-esteem (SE) at 12–13 years of age as a covariate *n* = 695**						
Gender Girls Boys	0.122 0	1.040	1.082	-0.11-0.35	0.002	
Mother’s educational level Post-secondary Secondary	0.071 0	0.514	0.264	-0.20–0.34	0.000	
Father’s educational level Post-secondary Secondary	0.139 0	1.025	1.050	-0.13-0.41	0.002	
The family’s economy worse medium high	0.488* 0.077 0	2.433 0.554	3.314	0.09–0.88 − 0.20–0.35	**0.009** 0.000	
SE at 12–13 years	0.095**	**8.951**	**(1,688)** **80.116**	**-0.12 – -0.07**	**0.104**	
						.14
**c) Both SE and MWB at 12–13 years of age as covariates n = 654**						
Gender Girls Boys	0.054 0	0.457	0.209	-0.18-0.28	0.000	
Mother’s educational level Post-secondary Secondary	0.097 0	0.709	0.503	-0.17–0.37	0.001	
Father’s educational level Post-secondary Secondary	0.146 0	1.084	1.175	-0.12-0.41	0.002	
The family’s economy worse medium high	0.381 0.069 0	1.891 0.506	1.940	0.02–0.78 − 0.20–0.34	0.006 0.000	
SE at 12–13 years	-0.057**	**4.361**	**(1,646)** **19.022**	**-0.08—0.03**	**0.029**	
MWB at 12–13 years	0.256**	**4.895**	**(1,646)** **23.962**	**0.15–0.36**	**0.036**	
						.18

*p* < .005 * *p* < .001**

Despite gender differences in the association with perceived MWB, no significant associations were detected in the statistical model. Therefore, the same analyses were carried out for girls and boys separately (Table 6).

**Table 6:** Covariates predicting perceived mental well-being (MWB) at age 17, separately for girls and boys

Covariates group	β	t	(df), F	Cl	η2	R^2^	Significant independent variables
**a) MWB at 12–13 years of age as a covariate *n* = 661**							
a) Girls *n* = 376	0.390**	7.427	(1,370) 55.150	0.287-0.493	0.13	.167	Mother’s education β = 0.395*
a) Boys *n* = 285	0.411**	5.612	(1,279) 31.496	0.267-0.555	0.10	.148	
**b) Self-esteem (SE) at 12–13 years of age as a covariate *n* = 695**							
b) Girls *n* = 399	− 0.107**	-8.185	(1,393) 66.994	− 0.132–0.081	0.15	.185	Mother’s education β = 0.382*
b) Boys *n* = 296	− 0.072**	-3.972	(1,290) 15.780	− 0.108–0.036	0.05	.103	Family´s economy β = 0.736*
**c) Both SE and MWB at 12–13 years of age as covariates *n* = 654**							
c) Girls *n* = 374 MWB SE	0.195* − 0.076**	2.882 -4.412	(1,367) 8.304 19.47	0.062-0.328 − 0.109–0.042	0.02 0.05	.209	Mother’s education β = 0.360*
c) Boys *n* = 280 MWB SE	0.331** **− 0.030**	3.941 -1.466	(1,273) 15.528 2.148	0.165-0.496 − 0.070-0.010	0.05 0.01	.150	

*p* < .005 * *p* < .001**

Covariates in the summary of the hierarchical ANCOVA analysis predicting perceived mental well-being (MWB) at age 17 (W5), separately for girls and boys.

Re-analysing the hierarchical ANCOVA separately for boys and girls showed differences between girls and boys. The outcome of MWB at the age of 17 depended more for girls on their mother’s education than for boys. For boys, a difference was present regarding SE as the sole covariate with a significant association for families’ economy, which was in line with results from the total sample in the ANCOVA models a and b (see Table 5). The overall effect on MWB at age 17 is more significant for girls than boys, independent of the model.

## Discussion

The study aimed to appraise and deepen the scientific understanding of adolescents’ self-reported self-esteem (SE) at age 12−13 from a resource perspective and test its ability to predict subsequent perceived mental well-being (MWB) at age 17. SE was significantly negatively associated with perceived MWB, explaining 18 per cent of the variance in a hierarchical ANCOVA model, including initial MWB as a covariate. For every additional point on the SE scale at age 12−13, adolescents rated their MWB at 17 years by 0.06 units less. Arguably, normal SE rather than high SE at 12−13 years is predictive of later mental well-being. The negative correlation may also be connected to girls’ lower initial SE than boys. The association was not surprising, though earlier research has reported associations between self-esteem and mental disorders [18]. However, the association with mental well-being in adolescence is not well represented in previous research, and several researchers argue for exploring this dimension in mental health [12].

Girls often report more internalising mental health problems, and gender differences have previously been shown [19]. In this study, however, gender did not stand out as an independent predictor of MWB at age 17 as expected. However, when conducting the analysis separately for boys and girls, the association between SE, initial MWB, and MWB at age 17 was stronger for girls than for boys. When first controlling for initial perceived MWB, it affected girls by a variance of 13 per cent, while it affected boys by only 10 per cent. The association was inversely significant when using initial SE as a covariate; girls showed three times higher eta square than boys (15% vs. 5%). SE as a predictor of future MWB has a more significant impact on girls than boys: therefore, supporting girls with low SE might be one way to support their future well-being.

Mental well-being is important for boys as well. In a school-based survey in Norway, a similar result was found regarding self-esteem, gender, and life satisfaction, where girls reported lower SE and boys had higher quality of life [30]. In our study using MWB at age 12−13 as a covariate in the model, initial MWB could explain four per cent of the variance and again SE three per cent of the variance in the model. When controlling for gender, female gender had a five per cent eta square effect on SE and only a two per cent effect on MWB. For boys, the situation was reversed (1% vs. 5%). One explanation could be that girls reported lower SE and MWB than boys. This reasoning aligns with results from a cohort study [15] following adolescents’ self-esteem and mental health during a school year. When these two estimates were combined, they highlighted the importance of handling questions like these from a gender perspective, especially in mental health. Low self-esteem for girls early in life and low mental well-being for boys need to be addressed.

In this study, self-esteem was stable with a slight decrease, while previous research has indicated that self-esteem increases with age [31]. However, four years may not be sufficient for this kind of assessment. Looking at the total sample’s SE, most adolescents had normal levels of SE. The prevalence of low self-esteem has been found twice as common in girls as in boys. Boys on the other hand more often had high self-esteem [20]. One explanation can be, as Agam et al. [32] explained in their study regarding gender roles in society and their influence on adolescents’ self-esteem, that it is not surprising that boys report higher self-esteem. Boys are more likely to be in situations encouraging power, excitement, competition, and conflict. Girls are more likely to encounter support, self-disclosure, and intimacy situations. Therefore, girls develop emotions related to internalising dimensions, while boys tend to build emotions related to externalising dimensions [32]. Global self-esteem, estimated by Rosenberg’s self-esteem scale, is influenced by others’ values of the individual [33]. Changes are common in the identification period during adolescence, from childhood to adulthood [34]. This identification connects to self-concept or self-esteem, and the present study consists of persons in adolescence, from 12−13 to 17 years of age. However, there is an ongoing debate on whether self-esteem is stable during a lifespan [35]. Kuster and Orth [36] found in their longitudinal lifelong follow-up study of participants aged 14 years and onwards that those with high self-esteem at a given time very likely also had high self-esteem one year later, as well as five, 10, and even 30 years later. These changes during adolescence also affected mental health, which is visible in our study. Regarding changes during adolescence, Patalay et al. [37] reported decreasing psychological well-being with increasing age, which may be an essential factor in social and academic stress and potentially affect self-esteem.

Thirty per cent of the adolescents with high self-esteem reported good mental well-being, while 16 per cent had low mental well-being. However, most had good mental well-being when looking at those with low self-esteem. However, in another study, boys seemed to have a higher mental well-being in both the young and older age groups [38]. A review [39] showed the complexity between self-esteem and mental health problems. Low self-esteem can predispose an adolescent to develop a poor mental well-being. Still, the opposite is also possible: Low self-esteem can be induced by poor mental well-being. In the review, eight out of the ten studies included low self-esteem in young people. It appears to be a relatively weak predictor of the development of anxiety and depression in later adolescence and young adulthood. On the other hand, adolescents with anxiety or depression disordersespecially those comorbid with these diagnoses, were likely to have low self-esteem. Kean and Loades [39] also found that adolescents with mental health problems had lower self-esteem than those without. This is in line with our longitudinal study, although most adolescents aged 12−13 showed average self-esteem and normal scores of perceived mental well-being. This knowledge was already on the agenda in the 1950s when Jahoda [21] developed the connection between mental health and attitudes towards oneself. In recent years, positive psychology has been discussed more in research [12], but there are still difficulties in dealing with different meanings of concepts concerning mental health [40].

Global self-esteem is connected to psychological well-being [33], and this study sought to estimate positive predictors of mental health from a salutogenic perspective. Mental health concerns coping with and handling different stressors during a lifetime. From a salutogenic perspective, this means perceiving the situation also as meaningful [3]. Some previous studies have focused on outcomes that describe positive mental health. In the Canadian COMPASS study (*n* = 74501) following students aged 12 to 19, Romano et al. [38] found a significant decrease in the mean of mental health (estimated by flourishing) from ninth to 12th grade (32.14–31.29). Therefore, a specific outcome variable, perceived mental well-being (MWB), was created for the study. The intention was to create a variable covering mental well-being in line with Westerhof and Keyes’s mental health continuum [17], arguing for exploring the well-being dimension within mental health. The mental health continuum measures three aspects of mental well-being: emotional, psychological, and social well-being. Two variables in this study, ‘How do you feel about life right now’ and ‘I think my life has purpose and meaning’ relates to psychological well-being, and the question ‘How healthy do you think you are?’ relates to emotional well-being. The created variable MWB does, in that sense, relate to mental well-being despite lacking a specific item measuring social well-being. The concept the variable refers to differs from subjective well-being, which usually focuses on quality of life [41] which again mostly focuses on happiness and life-satisfaction. In Keyes’ model, happiness, and life satisfaction relate primarly more to emotional well-being than the concept of mental well-being the present study does. The three questions applied in this study refer to a sense of health and purpose. Including a more psychological aspect of well-being does not only involve hedonistic but also eudaimonic aspects of well-being, and by that also a sense of purpose. While there is empirical evidence showing that happiness and life-satisfaction are associated [42] with better health this study focuses on mental well-being indicating that self-esteem affects mental health. The psychological dimension including purpose and meaning is a concrete perspective which is applicable in school to motivate adolescents to enhance their mental health.

A mother’s educational level was significantly associated with initial SE and MWB for girls, which aligns with [43] findings on children and adolescents of mothers with low levels of education who had significantly more mental health problems. In our study, 60 per cent had mothers with post-secondary education. When controlling for the family’s economy, there was a significant association with SE, especially in boys, in line with an earlier study [22]. The association with the mother’s educational level was more dominant among girls.

### Limitations

In all longitudinal studies, participant drop-out is an almost inevitable event, and at the same time, a longitudinal study design is a strength. In this study, the total population of 12−13-year-olds from four municipalities was invited, and the majority participated. However, in senior high school, at age 17, adolescents were more spread out and more difficult to contact, increasing the dropout rate. While 1472 participated in the first wave, only 779 participated in the fifth regarding SE vs. 1414 in the first wave and 746 in the fifth regarding mental well-being. Of these, only 654 responded to both waves and questionnaires. Looking at those participating in Wave 1 but not in Wave 5 vs. those participating in both, we see a somewhat higher attrition rate among boys. Regarding SE and MWB, a slight non-significant difference in the mean scores (*p* = .269 vs. *p* = .346) could be detected, pointing at a random dropout.

Another limitation is using data from already gathered survey responses (i.e., secondary analysis). The strength of obtaining ready-collected longitudinal data is hampered by what variables were included and when the data related to them were collected. An outcome variable had to be developed, which was not validated as a scale in other studies. Theoretically and also according to a statistical evaluation, these variables fit together. The questionnaire lacked a specific item measuring social well-being as the third aspect of the mental health continuum, which may, besides the regression to the mean phenomenon, have impacted the negative association between initial self-esteem and subsequent mental well-being in the ANCOVA models. However, when creating the variable, the aim was to assess similar content, as is the case concerning the well-validated scale health continuum created by Westerhof and Keyes [12]. Another aspect related to when data on SE was collected was that the data was available only from waves 1 and 5. However, a strength worth mentioning is the salutogenic approach to estimating predictors of positive mental health rather than identifying predictors of mental health problems or mental diagnoses, as most previous studies have done.

## Conclusions

Good self-esteem in early adolescence predicts a continuation of a favourable situation and increases the probability of subsequent good mental well-being. Adolescents’ mental well-being explains their self-esteem by 18 per cent, especially among girls. This highlights the question of sophisticated measuring instruments from a gender and resource perspective. Other researchers have also focused on these methodological aspects, which supports further research on handling the complex mental health situation. Accordingly, supporting self-esteem early in life can promote mental well-being.

### Supplementary Information

**Additional file 1.** Table of Flowchart within LoRDIA regarding self-esteem (SE) and mental well-being (MWB)

## Data Availability

The datasets used and/or analysed during the current study are available from the corresponding author on reasonable request.

## Abbreviations

LoRDIA: Longitudinal Research on Development In Adolescence
MWB: Mental well-being
SE: Self-esteem
WHO: World Health Organization
