# Supplementary material for: The association between adolescents’ self-esteem and perceived mental well-being in Sweden in four years of follow-up
Source: BMC Psychol. 2023 Nov 25;11:413. doi: 10.1186/s40359-023-01450-6 (PMC10676579; doi:10.1186/s40359-023-01450-6)
Supplement: Supplementary file 1 — Additional file 1. Table of Flowchart within LoRDIA regarding self-esteem (SE) and mental well-being (MWB) [file 40359_2023_1450_MOESM1_ESM.docx]

**Table of Flowchart within LoRDIA regarding self-esteem (SE) and mental well-being (MWB)**

| **Wave (W)** | **Total invited** | **Study population** | **Agreed to participate** |  | **SE** | **MWB** | **SE+MWB** |
| --- | --- | --- | --- | --- | --- | --- | --- |
| **W1** | 2108 | 1790 | 1515 (85%) |  | 1472 #  g: 51%  b: 49% | 1414 # g: 51%  b: 49% |  |
|  |  |  |  |  |  |  |  |
| **W2** | +42 | 1884 | 1454 (77%) |  |  |  |  |
|  |  |  |  |  |  |  |  |
| **W5** |  | 1884 | 949 (50%) |  | 936 ###  g: 56%  b: 44% | 948 #  g: 56%  b: 44% |  |
|  |  |  |  |  |  |  |  |
| **Pair W1+W5** |  |  |  |  | 779 ## | 746 ## |  |
| **W1+W5** |  |  |  |  |  |  | 654 ####  g: 57% b: 43% |

g. = girls, b. = boys

**Presented in different tables in the manuscript:**

# = Table 1, Table 3

## = Table 2

### = Table 4

#### = Table 5, Table 6
